# The Ecological Mechanism of Coral–Algal Phase Shifts: A Case Study of Wenchang in Hainan Province

**DOI:** 10.1002/ece3.72746

**Published:** 2026-02-01

**Authors:** Yihua Lyu, Yangmei Zhang, Yuqian Li, Youqi Wang, Zhiqiang Chen, Yingyi Huang, Tuanjie Li

**Affiliations:** ^1^ Nansha Islands Coral Reef Ecosystem National Observation and Research Station Guangzhou China; ^2^ South China Sea Ecological Center, Ministry of Natural Resources Guangzhou China; ^3^ South China Sea Institute of Oceanology, Chinese Academy of Sciences Guangzhou China; ^4^ College of Hydrology and Water Resources Hohai University Nanjing China

**Keywords:** coral reef, coral‐algal interactions, *Lobophora*, macroalgae, phase shifts

## Abstract

The interaction between corals and algae is of great significance in maintaining the health of coral reef ecosystems. Globally, tropical coral reefs are being degraded by human activities, resulting in the decline of reef‐building corals and an increase in macroalgae. The study aims to determine the ecological mechanism of coral–algal phase shifts occurring in the coastal waters of Wenchang in Hainan Province, which are affected by human activities. The field survey results revealed that 177 species of reef‐forming corals belonged to 49 genera and 18 families, which were dominated by coral taxa *Favites*, *Porites*, *Galaxea*, and *Montipora*. Furthermore, there existed obvious competition between dominant corals and algae, especially *Lobophora*, which showed stronger competitive advantages compared to other algae. The salinity, NO_2_^−^, and NH_4_^+^ were the key environmental drivers that affected macroalgal abundance. There was a negative correlation between macroalgae and live coral cover and a positive correlation with coral mortality. Notably, the dominant algal species *Lobophora* had a significant negative correlation with the dominant coral taxa *Favites*, *Galaxea*, and *Montipora*. Further correlation analysis showed that significant interspecific competition existed among corals and algae themselves, which may function as the internal driving factor for the phase shifts of coral–algal relationships. These results will help us understand the role of different functional groups of algae in the degradation of coral reef ecosystems and lay the research foundation for the development of scientific and rational coral reef protection strategies.

## Graphical abstract

This study selected the coral reef areas in the coastal waters of Wenchang, Hainan, China, which were significantly affected by human activities. Through four consecutive years of field investigations, the key driving factors and potential mechanisms of ecological phase shifts between corals and macroalgae were identified, thus providing a scientific basis for strengthening coral reef management under the influence of human activities.

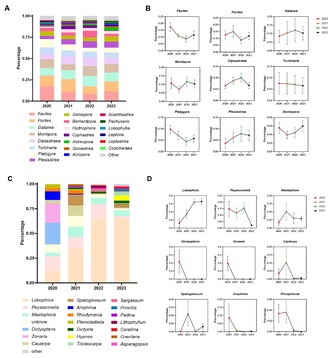

## 1 Introduction

Benthic algae are the main source of primary productivity in coral reef ecosystems, playing an important role in maintaining their stability. Competition is an important process in determining the structure and composition of benthic communities in coral reefs (McCook 2001). Particularly, the phenomenon of competition between corals and benthic algae is regarded as important for the overall health of coral reef ecosystems, especially during “phase shifts,” that is, the reef area changes from being dominated by corals to being dominated by macroalgae (Done 1992; Hughes 1994; Littler and Littler 1984; Miller 1998). It has been shown that competition between corals and macroalgae is a critical step during the degradation of coral reef ecosystems (McCook 1999; Miller and Hay 1998). However, coral reef ecosystems worldwide have been greatly degraded in recent decades due to the influence of human activities and global climate variability, and the coverage of living corals has declined sharply. In contrast, the coverage and biomass of macroalgae in reef regions has shown a rapid increasing trend (Steneck et al. 2004). The degradation of coral reefs has seriously affected the habitats of reef‐dwelling organisms and their biodiversity (Knowlton and Jackson 2008). In general, the overgrowth of macroalgae is regarded as indicative of degraded reef ecosystems, while the high coverage of hard corals indicates healthy reefs. According to the most recent assessment by the Intergovernmental Panel on Climate Change, 70%–90% of coral reefs worldwide will disappear by 2030 due to factors such as global warming (Hughes et al. 2019).

Three main competitive mechanisms between corals and macroalgae are generally recognized. First, the physical mechanism of the competition between corals and macroalgae primarily refers to the process of macroalgae directly or indirectly affecting the growth and recovery of corals through physical methods (McCook 2001). The physical mechanisms of competition between corals and macroalgae mainly include the covering effect caused by the overgrowth of macroalgae, the shielding effect formed by the canopy of macroalgae, the bruising effect caused by macroalgae on corals, and the priority preoccupation of space by macroalgae (McCook 2001). Second, the chemical mechanism of competition between corals and algae mainly refers to a chemical process in which allelopathic substances (fat‐soluble or water‐soluble organic secondary metabolites) produced by algae enter coral tissue and cause adverse effects on corals (Rasher and Hay 2010; Smith et al. 2006). Coral diseases are usually caused by microorganisms that may poison coral health (Bruno et al. 2007). The third is the microbial mechanism, which is an indirect way for algae to affect coral growth through microorganisms. Macroalgae can interfere with the microbial community on corals by releasing chemicals, including soluble organic matter (SOC) or allelochemicals, which is believed to increase the activity of microorganisms on corals and cause coral diseases (Smith et al. 2006). However, the above mechanisms have not been fully explored, and further research is needed to understand the competing relationships between corals and microalgae.

The benthic algae in coral reef ecosystems are divided into three functional groups: turf algae, macroalgae, and crustose coralline algae. In addition, the competition between corals, turf algae, and macroalgae is considered one of the most typical competitive relationships in coral reef ecosystems (McCook 2001). With the decline of live coral coverage, the overgrowth of turf algae and macroalgae has hindered the growth, reproduction, and recovery of corals, thus replacing corals as the dominant species. Turf algae and macroalgae grow fast, but corals are usually dominant in healthy coral reef ecosystems. However, field survey results have shown that the dominant position of most coral communities in the South China Sea has been replaced by benthic algae, among which turf algae and macroalgae have the most significant impact on coral reef ecosystems. In addition, macroalgae have become dominant on many remote reefs of the Xisha and Zhongsha Islands that corals once dominated (Chen et al. 2019). It can be seen that turf algae and macroalgae are the main sources of competitive pressure for corals in the South China Sea.

However, the direct experimental evidence of studies on the interaction between corals and macroalgae in reef regions is still lacking, and most of the existing evidence is only relevant or indirect (McCook 2001). It is unclear whether the growth of algae is the direct cause of the decline in coral cover (Aronson and Precht 2006). However, it is undoubted that the massive growth of algae will crowd out the ecological space of corals and affect the photosynthesis of the symbiotic zooxanthellae of corals (Titlyanov et al. 2007). In coral reef ecosystems, macroalgae vary in space and time owing to a combination of biological and abiotic factors (Brown et al. 2018; Diaz‐Pulido et al. 2007; Littler et al. 2006). Human activities, such as clearing land and fertilizing for agriculture, and the reduction of herbivores due to overdevelopment or heat stress, can upset the natural balance, resulting in fewer reef‐building corals and increased macroalgae cover (De'ath and Fabricius 2010; Diaz‐Pulido et al. 2009).

The increase in the abundance of macroalgae will lead to the enhancement of coral‐algal competition (Connell et al. 2004; Haas et al. 2009), which may play an important role in the degradation of coral reefs (McCook 1999). The effect of human disturbance and other factors on the competition between corals and macroalgae cannot be ignored. Nonetheless, less attention has been paid to the factors that cause competition between macroalgae and coral communities. In particular, the scientific explanation of the ecological mechanism that causes the phase shifts between macroalgae and coral communities is not deep enough. Therefore, the author conducted a continuous field survey of coral reef ecosystems in the Wenchang Coastal waters of Hainan, China, from 2020 to 2023. By analyzing the dynamic changes of coral‐algal community structure and its key driving factors, it is found that the interactions between corals and algae, corals and corals, and algae and algae are important driving factors to reveal the coral‐algal phase shifts. The results will help to evaluate the ecological role of macroalgae in the process of coral reef degradation and recovery and also facilitate an understanding of the health status of coral reef ecosystems, thus providing a theoretical basis and decision‐making reference for further strengthening the protection and management of coastal coral reef ecosystems in China.

## 2 Materials and Methods

### 2.1 Sample Collection

**FIGURE 1:**
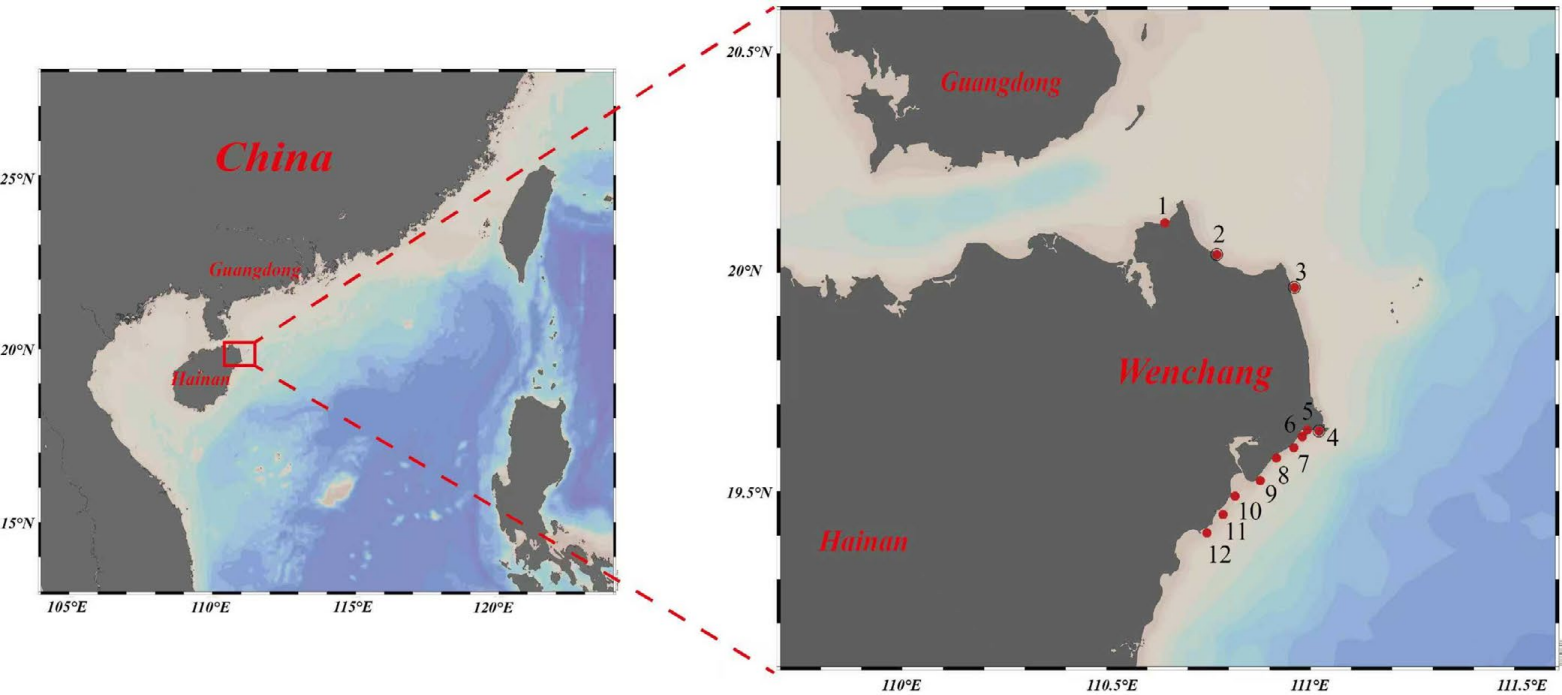
Sites of the coral reef ecosystem survey in Wenchang of Hainan province. (The surveyed sites are denoted by the red dot).

A field survey of the coral reef ecosystem was conducted in the Wenchang of Chinese coastal waters, which is located in the northeastern part of Hainan Island, between 19°21′ and 20°01′ north latitude and 110°28′ and 111°03′ east longitude. It borders the South China Sea to the east and southeast, and the Qiongzhou Strait to the north. The 12 specific surveyed sites are shown in Figure 1 and detailed sampling information for each station was supplemented (Table S1). The distribution characteristics of coral communities, reef‐dwelling organisms, reef habitats, and human activities in the investigated area are all included in site selection. Sample collection continued for 4 years, starting in 2020 and ending in 2023, which was carried out during the period from July to October every year.

### 2.2 Ecological Investigation of Coral Reefs

The field survey was carried out between July to October during 2020 to 2023, and each sample sites was observed once a year. All survey elements related to coral ecosystems were conducted according to the Technical Guideline for Investigation and Assessment of Coastal Ecosystems—Part 5: Coral Reefs (T/CAOE 20.5‐2020). The investigation of coral reefs was carried out by the cross‐sectional spline method. Three transects of different depths (3, 6, and 9 m) were established at each location, while SCUBA diving was carried out. Each transect spanned 50 m and was positioned in parallel using a tapeline. The details on the sampling protocol of each sites are listed (Table S1). For the analysis of coral communities, standardized procedures were adopted to capture point intercept transect (PIT) videos (Hill and Wilkinson 2004). A survey technician carried a handheld camera, with the lens 0.2–0.3 m away from the transect tape, swimming slowly and uniformly along the belt from its start point. The camera was directed vertically downward, focused on the tape to clearly record the organisms and substrate. The recording time was for at least 10 min until reaching the end of the tape. Another technician took macro photos of various types of corals under the tape after the video recording and collected a small number of rare species specimens to assist in species identification. Coral sample identification is carried out by professional technicians with coral identification experience based on morphology. Randomly, 40 quadrats measuring 25 × 25 cm were positioned along the reef within 2.5 m of each tape side to take sample photos of each quadrat to record hard coral recruitment. And the quadrat was randomly arranged to avoid being artificially placed in areas with a high concentration of sand, coral, algae or other organisms. These quadrats will contain newly born young corals and some reef‐dwelling organisms. By interpreting the number of newly born corals in the sample boxes, the coral's recovery ability can be characterized. The survey of coral reef fish was conducted using the tape section video recording method described by Technical Guideline for Investigation and Assessment of Coastal Ecosystems—Part 5: Coral Reefs(T/CAOE 20.5‐2020). After the sample tape is laid out, wait for 10 min. Then, a survey technician held a camera, about 0.5 m above the bottom, and started to move slowly and uniformly along the tape from the starting point. The camera lens was held horizontally and aimed forward along the tape, with the focal length set at approximately 35 m to make sure that the fish within 2.5 m on both sides of the sample band in the field of view can be captured. Continue filming until the end point of the transect tape, with the total video duration being at least 5 min. Other personnel should avoid appearing in front of the camera during filming. Simultaneously, another survey technician followed behind the videographer to take macro photographs of fish on the transect and its sides, which will serve as Supporting Information for identification and analysis. In addition, 40 quadrats of 25 × 25 cm were randomly positioned within a 2.5 m range on both sides of the transects to capture photographs of various benthic macroalgae at a macro distance. And algae samples were also collected if needed. The identification of algae samples is carried out by professional technicians with experience in algae identification based on morphology.

### 2.3 Water Sample Collection

Water samples were collected at 12 locations in Wenchang, with a total of 41 water samples collected. Water temperature (Tem), salinity (Sal), transparency (Tra), water depth (WD), pH, dissolved oxygen (DO), dissolved inorganic nitrogen (encompassing NH_4_^+^‐N, NO_2_^−^‐N, and NO_3_^−^‐N), inorganic phosphate (PO_4_^3−^‐P), total nitrogen (TN), total phosphorus (TP), chlorophyll a (Chla), chemical oxygen demand (COD), suspended solids (SS), and substrate were detected as water parameters. The collection, storage and transportation were carried out in accordance with the Specification for Oceanographic Survey (GB/T 12763‐2007). The water samples were filtered through an acetic acid membrane filter (0.45 μm pore size) and collected into glass bottles, which were stored at −20°C for later analysis. The concentrations of dissolved inorganic nutrients (nitrate, nitrite, ammonium, phosphate, and silicate) were detected by Segmented Flow Analysis method via the SEAL Analytical AA500 auto‐analyzer system. The analysis was conducted by colorimetry in accordance with Code of practice for marine monitoring technology Part 1: seawater (HY/T 147.1‐2013) and Technical specification for offshore environmental monitoring Part III: offshore seawater quality monitoring (HJ 442.3‐2020). The concentration of dissolved inorganic nitrogen (DIN) was calculated as the sum of nitrate, nitrite, and ammonium. A thermohalimeter was used to assess seawater temperature and salinity. The pH value was measured with a pH meter. SS samples were dried and weighed using a scale. The DO and COD values were determined respectively by the Winkler titration method and the alkaline potassium permanganate method according to the Specification for oceanographic survey—Part 4: Seawater analysis (GB/T 17378.4‐2007). The Chla values were determined by fluorescence spectrophotometry according to the Specification for oceanographic survey—Part 6: Marine biological survey (GB/T 12763.6‐2007).

### 2.4 Ecological Statistical Analysis

Figures and maps of sample locations were visualized using Ocean Data View 5.6.1 software. Graphs were processed in GraphPad Prism (Version 10.1.2), and the *t*‐test was used for comparison between the two groups. Statistical analyses were conducted in R 4.4.1 interfaced with RStudio 2024.09.0‐375. Redundancy analysis (RDA) was used to explore the relationship between coral and algae communities and if the detrended correspondence analysis ranking axis values were < 4. RDA was performed by packages “vegan” and “ggplots.” The species diversity (alpha diversity) was also conducted by packages “vegan.” The corr.test from packages “Psych” was used to investigate all pairwise relationships between corals, algae, and environmental factors. Spearman analysis and FDR were selected to calculate the R‐value and P‐value of all relationships. Packages “ggplot” were used for heatmap visualization of all pairwise correlations between corals, algae, and environmental factors. The visualization and beauty of the heatmap were generated using Adobe Illustrator 2020.

## 3 Results

### 3.1 Species Diversity of Corals and Algae

**FIGURE 2:**
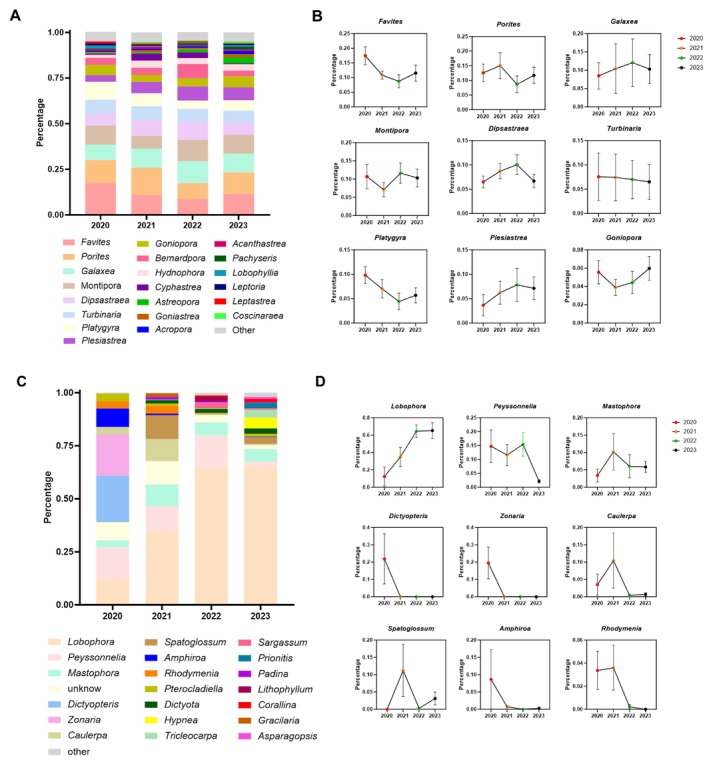
The species diversity of corals (A, B) and algae (C, D) in Wenchang from 2020 to 2023 at the genus level. The error bars represent the standard error of the mean (SEM) in (B, D).

In this study, we investigated the coral community in Wenchang from 2020 to 2023. Our study found 177 scleractinian coral species belonging to 49 genera and 18 families. The information on the species diversity of corals and algae at each station was supplemented (Table S2). At the genus level, the *Favites*, *Porites*, *Galaxea*, *Montipora*, *Dipsastraea*, *Turbinaria*, *Platygyra*, *Plesiastrea*, and *Goniopora* corals were dominant, and the total proportion was more than 75% (Figure 2A; Table S3). The abundance of *Favites*, *Turbinaria*, and *Platygyra* corals generally showed a downward trend (Figure 2B; Table S3), while the abundance of *Galaxea*, *Plesiastrea*, and *Goniopora* corals generally showed an upward trend over the past four years (Figure 2B; Table S3). At the same time, the abundance of *Porites*, *Montipora*, and *Dipsastraea* corals showed a fluctuated tendency from 2020 to 2023 (Figure 2B; Table S3). In terms of algal species diversity, the eight dominant algae genera were *Lobophora*, *Peyssonnelia*, *Mastophora*, *Dictyopteris*, *Zonaria*, *Caulerpa*, *Spatoglossum*, and *Amphiroa*, accounting for > 80% of the composition ratio in all surveyed sites (Figure 2C; Table S4). When compared to 2020, the proportion of *Lobophora*, *Mastophora*, and *Spatoglossum* increased, and the average proportion of *Lobophora* increased by 65% in 2023 (Figure 2D; Table S4). The proportion of *Peyssonnelia*, *Dictyopteris*, *Zonaria*, *Caulerpa*, and *Amphiroa* was reduced in 2023 (Figure 2D; Table S4).

### 3.2 The Structural Characteristics of Coral and Algae Communities

**FIGURE 3:**
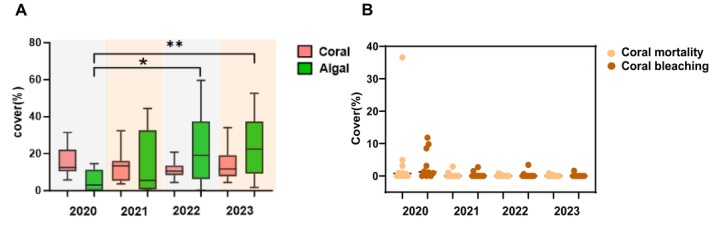
Coverage of coral and algae in Wenchang from 2020 to 2023 (A). The scatter plot of coral mortality and coral bleaching in Wenchang from 2020 to 2023 (B).

The investigation showed that the coverage of live coral was 16.19% in 2020. Still, the coverage of live coral declined to 12.96%, 10.94% in 2021, 2022, respectively, but in 2023, the coverage of live coral increased compared to 2022 (Figure 3A; Table 1). In 2020, the coral mortality was 4.70%. In 2021, 2022, and 2023, the coral mortality was 0.33%, 0.11%, and 0.33%, respectively, with the coral bleaching percentage also being the highest in 2020 (3.66%; Table 1).

**TABLE 1:** Main indicators for the coral reef community in Wenchang from 2020 to 2023.

Indicators	2020	2021	2022	2023
Live coral cover (%)	16.19	12.96	10.94	13.95
Croal recruitment (ind/m^2^)	1.09	2.01	1.38	0.98
Croal mortality (%)	4.70	0.33	0.11	0.33
Croal bleaching (%)	3.66	0.35	0.13	0.13
Macroalgae cover (%)	5.01	15.09	21.63	22.95

The average hard coral recruitment in Wenchang was lower than 3 ind/m^2^ from 2020 to 2023, indicating that these regions had relatively poor potential for restoration (Table 1). Furthermore, the coverage of macroalgae was also investigated, and its coverage was at its lowest level in 2020 (5.01%), whereas the coverage of macroalgae suddenly increased to 15.09% in 2021. Additionally, the coverage of macroalgae continued to rise in 2022 and 2023, along with coral cover, which continued to decline till 2022 but increased in 2023. Furthermore, the scatter plot between coral mortality and bleaching showed that the trend of coral bleaching and coral mortality is consistent (Figure 3B; Table 1). It is worth noting that coral bleaching was the most serious in 2020, and coral mortality was also highest in 2020. In 2020, a massive coral bleaching event ever occurred near the coast of Hainan Island. The massive coral bleaching led to a large number of coral mortalities.

### 3.3 The Key Environmental Drivers Affecting Corals and Algae

**FIGURE 4:**
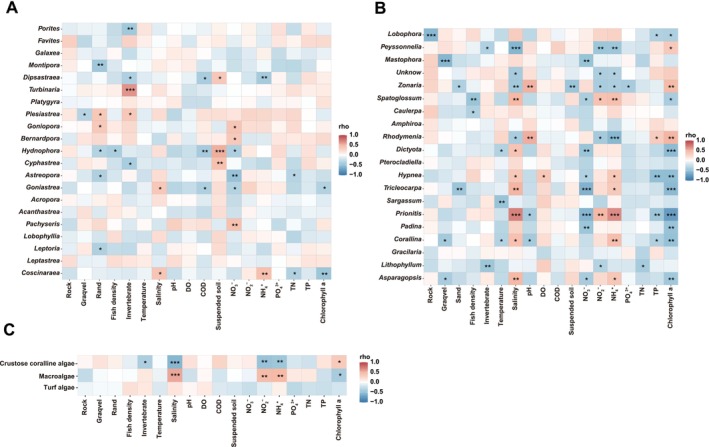
Heat maps show Spearman's relationship between environmental factors and coral species (A), algal species (B), and three functional groups (including crustose coralline algae, macroalgae, and turf algae) of algal species (C), respectively. The P‐values of correlation coefficients were corrected using the BH method (“*” represents *p* < 0.05, “**” represents *p* < 0.01, “***” represents *p* < 0.001).

The relationship between environmental factors and dominant coral species was analyzed, with the total proportion more than 95%. Of these, *Porites* and *Dipsastraea* exhibited a noteworthy negative correlation with invertebrates, while *Turbinaria* and *Plesiastrea* showed a strongly positive correlation with invertebrates (Figure 4A; Table S5). Furthermore, *Montipora* showed a significant negative correlation with sand, but *Plesiastrea* and *Goniopora* had a strongly positive correlation with sand (Figure 4A; Table S6). Additionally, *Dipsastraea* had a negative correlation with COD and NH_4_^+^ while showing a positive correlation with suspended soil (Figure 4A; Table S6).

The correlation between environmental factors and algal species was also analyzed. Salinity, NO_2_^−^, and NH_4_^+^ strongly negatively affected *Peyssonnelia* and *Zonaria* but positively affected *Spatoglossum* (Figure 4B; Table S7). Of which, the NO_3_^−^ significantly negatively influenced *Mastophora* and *Spatoglossum* (Figure 4B; Table S7). Additionally, Chla was negatively correlated with *Lobophora* and *Spatoglossum* but positively correlated with *Peyssonnelia* and *Zonaria* (Figure 4B; Table S7). The total phosphate demonstrated a strong negative correlation with the most dominant algal species, *Lobophora* (Figure 4B; Table S7).

Our results showed that the functional group of crustose coralline algae had a significant negative correlation with invertebrate, salinity, NO_2_^−^, and NH_4_^+^, but had a strongly positive correlation with Chla (Figure 4C; Table S8). While the functional group of macroalgae was strongly positively affected by salinity, NO_2_^−^, and NH_4_^+^ and negatively affected by Chla (Figure 4C; Table S8).

### 3.4 The Coral–Algal Interactions

**FIGURE 5:**
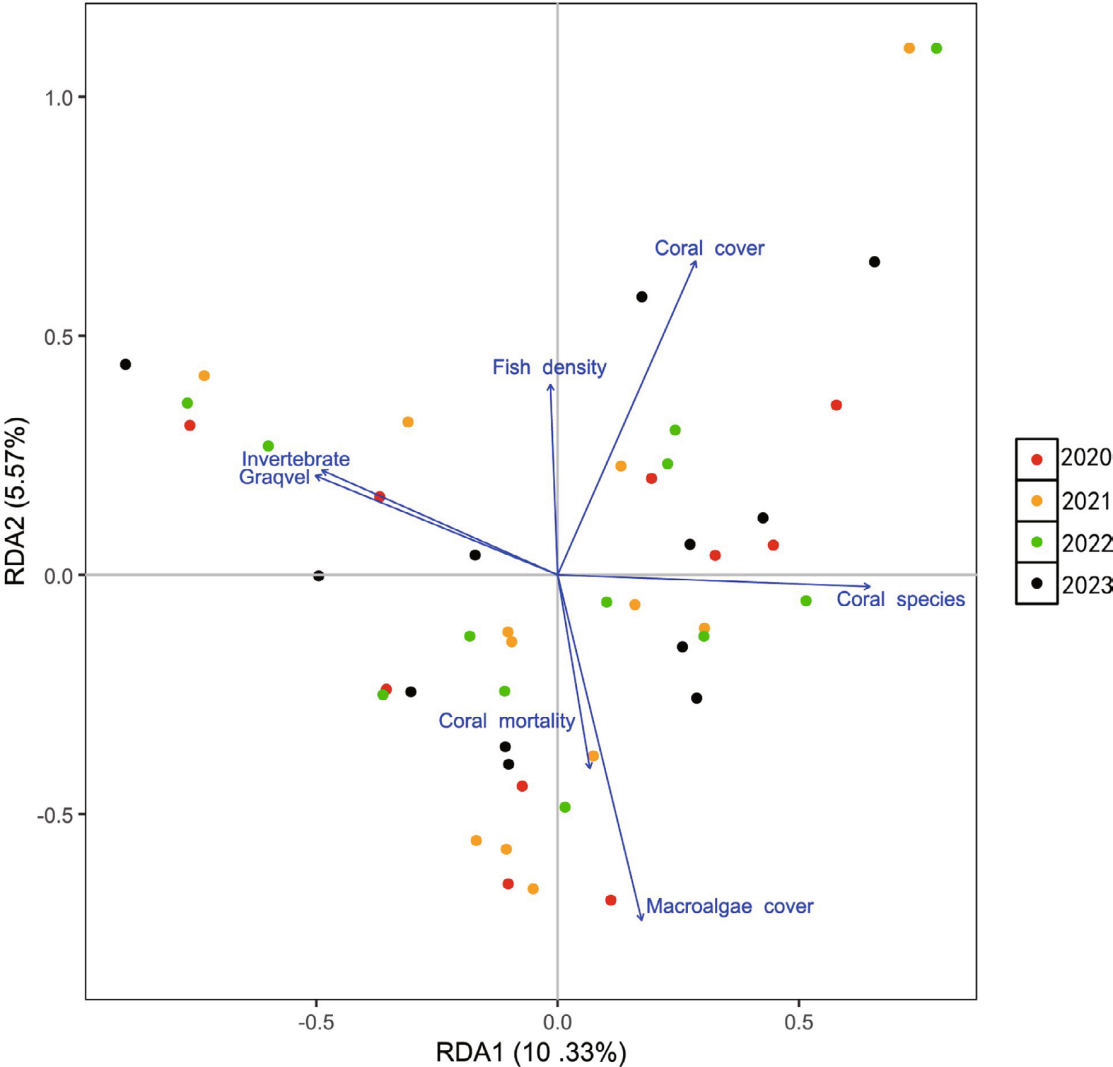
Transformation‐based redundancy analyses (tb‐RDA) illustrate the effects of significant drivers for the coral reef community (Type 2 scaling with *R* ^2^ = 0.223 and *p* = 0.001; *p*‐value of RDA1 = 0.003 and RDA2 = 0.047).

**FIGURE 6:**
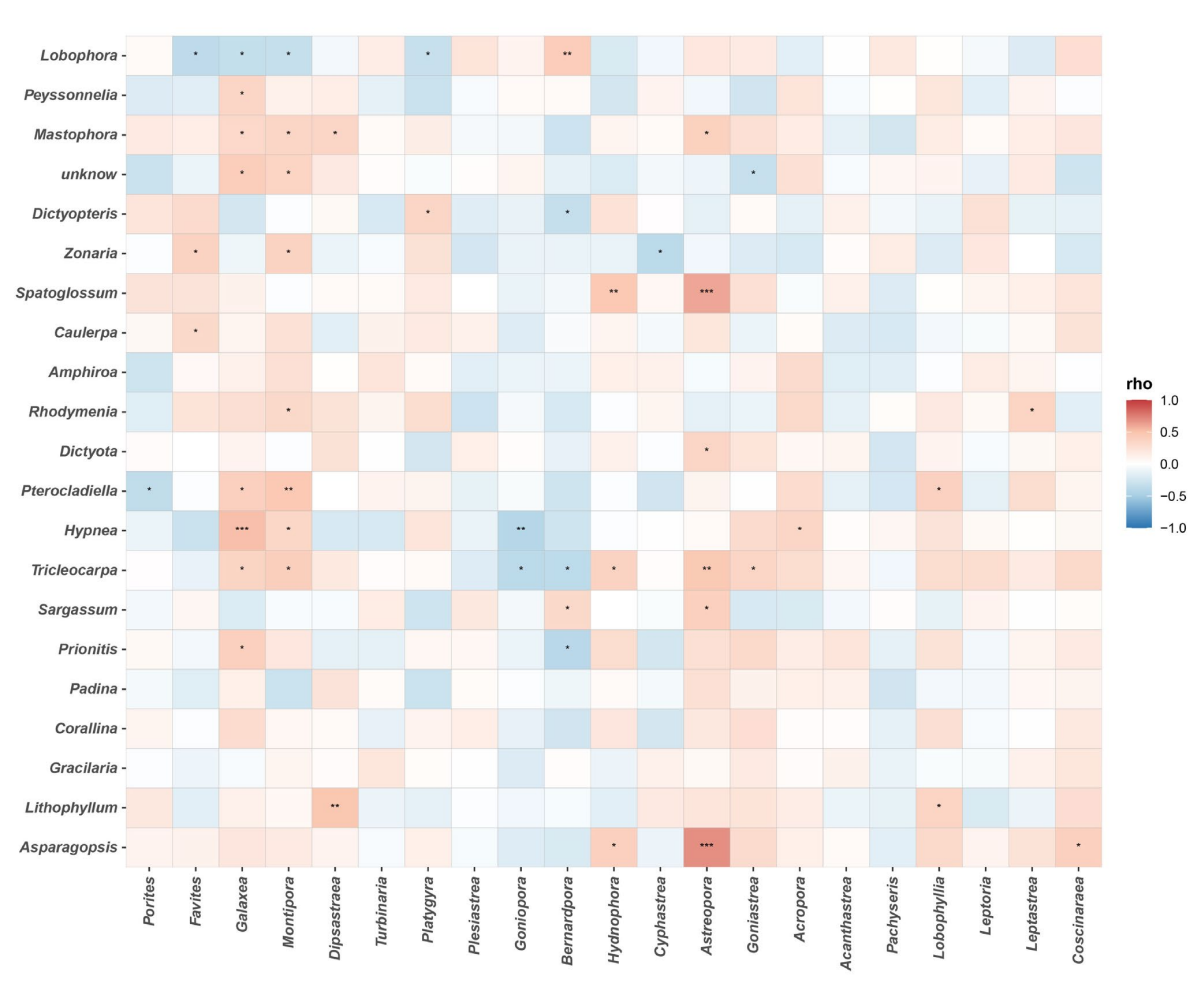
Heatmaps show Spearman's relationship between coral and algal species. The P‐values of correlation coefficients were corrected using the BH method (“*” represents *p* < 0.05, “**” represents *p* < 0.01, “***” represents *p* < 0.001).

In the RDA analysis, coral cover displayed a positive correlation with fish density (Figure 5). Remarkably, RDA underscored a negative correlation between coral cover and macroalgae (Figure 5). Further analysis through Spearman's relationship indicated that the coral species were associated with different functional groups of algal species. The most dominant algal species, *Lobophora*, had significantly negative effects on the dominant coral species *Favites*, *Galaxea*, *Montipora*, and *Platygyra* (Figure 6; Table S9). At the same time, other dominant algal species (e.g., *Peyssonnelia*, *Mastophora*, *Dictyopteris*, *Zonaria*, and *Caulerpa*) showed a significantly positive correlation with other dominant corals (Figure 6; Table S9).

**FIGURE 7:**
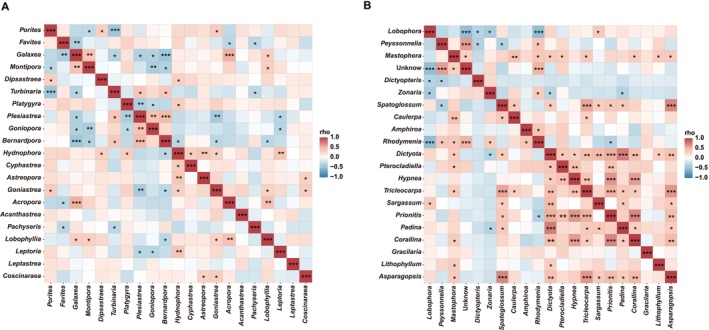
Heatmaps show the Spearman's relationship among different coral species (A) and different algal species (B). The P‐values of correlation coefficients were corrected using the BH method (“*” represents *p* < 0.05, “**” represents *p* < 0.01, “***” represents *p* < 0.001).

Additionally, the correlation between the dominant algae and coral species was also analyzed. The results showed that *Favites* had a significant negative correlation with *Galaxea* (Figure 7A; Table S10). The *Porites* had significant negative correlations with *Montipora* and *Turbinaria* but positive correlations with *Dipsastraea* (Figure 7A; Table S10). *Galaxea* strongly negatively affected *Turbinaria*, *Plesiastrea*, and *Goniopora* but positively affected *Montipora*. Among algal species, the dominant algal species, *Lobophora*, significantly negatively influenced *Dictyopteris*, *Zonaria*, and *Rhodymenia* (Figure 7B; Table S11). *Peyssonnelia* was strongly negatively influenced by *Dictyopteris* and *Spatoglossum* but positively influenced by *Rhodymenia* (Figure 7B; Table S11). The *Mastophora* was strongly positively affected by *Caulerpa*, *Rhodymenia* (Figure 7B; Table S11).

## 4 Discussion

### 4.1 Succession Characteristics of Coral and Algal Community Structures

It can be seen that the coral and algal community structures showed obvious succession characteristics, among which the coral taxa *Favites*, *Porites*, *Galaxea*, and *Montipora* were dominant. In contrast, the dominant algal species, *Lobophora*, showed a strong competitive advantage over other algal taxa. The functional group of macroalgae represented by *Lobophora* genus can cause damaging effects on corals through direct contact (Inagaki and Longo 2024). The coverage analysis of coral and algae showed that the live coral cover has declined, corresponding to the increase in macroalgae from 2020 to 2022, indicating that the dominant taxa have altered from corals to macroalgae. The RDA analysis has further verified that the increase in macroalgae will cause damage to the growth of corals. Moreover, the macroalgae cover is negatively correlated with the invertebrates and fish, indicating that macroalgae growth may be affected by the reduction of predators, which is similar to some previous studies that the decrease in herbivore fishes may facilitate algae growth as a result of overfishing (Littler and Littler 2006; McCook 1999; Smith et al. 2014). Human activities such as aquaculture, fishing, and sewage discharge are the main sources of pressure affecting the growth and development of corals along the coastal waters of Wenchang. In the last 10 years, marine fishery activities and coastal tourism in Wenchang City have increased year by year, and the pressure on the growth of coral reefs along the Wenchang coastal waters has gradually increased.

### 4.2 The Key Environmental Drivers of Coral–Algal Phase Shifts

The analysis results based on Spearman's correlation indicated that it is not convincing to focus solely on the correlation between the dominant coral or algal taxa and environmental factors, while more attention should be paid to the macroalgae that interact with corals.

According to the literature described, reef benthic algae were divided into three functional groups: crustose coralline algae, macroalgae, and turf algae (Barott and Rohwer 2012; Barott et al. 2012; Brown et al. 2017). We found that the functional group of macroalgae was positively affected by salinity, NO_2_^−^ and NH_4_^+^ but negatively affected by Chla, revealing that the input of nutrients may encourage the growth of macroalgae, which is similar to what some lectures have described. The distribution and concentration of Chla are related to the presence of phytoplankton. The phytoplankton biomass was identified by measuring Chla (Marlian et al. 2015). Still, since macroalgae had the advantage of competing with phytoplankton for a limited ecological niche, it caused a low Chla concentration. In contrast, macroalgal abundance tended to increase in the studied region. Studies have demonstrated that salinity can affect the growth of phytoplankton (Sew and Todd 2020). Rapid and large salinity changes will keep algal cells in a state of osmotic stress, thus disturbing cellular homeostasis (Lionard et al. 2005).

In our study, the salinity, NO_2_^−^, and NH_4_^+^ were the key environmental drivers affecting macroalgal abundance. At the same time, some studies have also described that variations in temperature and light play a key role in the abundance of tropical macroalgae (Brown et al. 2018). According to the results of the previous studies, salinity was positively correlated with coral reef diversity and recruitment (Lyu et al. 2024), so further work should be conducted to explore the extent to which the threshold of salinity is reached to be suitable for macroalgal growth. Moreover, the functional group of crustose coralline algae can facilitate the recruitment of coral larvae and the development of a reef framework (Gómez‐Lemos et al. 2018; Harrington et al. 2004), which had a significantly negative correlation with salinity, NO_2_^−^, and NH_4_^+^ and a strongly positive correlation with Chla. Studies have shown that the positive impact on coralline algae only occurs under high‐level herbivorous conditions. In the absence of herbivorous fish, increasing nutrition will not be beneficial to the growth of coralline algae (Burkepile and Hay 2006). This can be explained by the surveyed results that the coral reef fish in the Wenchang Sea region were mainly non‐herbivorous fish.

The dominant algal species *Lobophora*, classified as macroalgae, has increased sharply from 2020 to 2023, corresponding to *Peyssonnelia*, which has declined from 2020 to 2023, suggesting that macroalgae have a stronger competitive advantage in the interaction with corals. Environmental factors may act as the key drivers affecting competition among algal species, reflecting the dynamic process of macroalgae gradually replacing corals as the dominant taxa.

### 4.3 Interspecific Competition Among Coral and Algal Species

As we can see from the species diversity of the coral reef community structure, there was an obvious interspecific competition between coral and algal species. In terms of coral taxa, the most abundant species of reef‐building corals in the surveyed area were *Favites*, *Porites*, *Galaxea*, and *Montipora*, dominated by massive corals. While the branching corals *Acropora* have been found, they were rare in number and only distributed in shallow water at some stations. Compared with branching reef‐building corals, massive corals belong to a wide ecological niche and have better adaptability to harsh environments (Li et al. 2012; Zhou et al. 2017). Owing to the interspecific competition for a limited ecological niche, the dominant corals have been altered. In terms of algal species diversity, the phenomenon of interspecific competition was particularly significant. In particular, the algal species *Lobophora*, which can cause detrimental effects on coral‐associated microbial communities and early life stages (Morrow et al. 2017), has become dominant, thus resulting in the decline of other algal taxa such as *Peyssonnelia*, *Dictyopteris*, *Zonaria*, *Caulerpa*, and *Amphiroa*. The interspecific competition between corals and algae themselves acts as the internal driver that affects coral–algal interactions. Algae with different properties have different effects on corals, which may influence the outcome of the coral‐algal interactions.

### 4.4 Interactions Between Corals and Algae

Based on the correlation analysis results of dominant corals and algae, it is clear that the most dominant algal species, *Lobophora*, had significantly negative effects on the dominant corals *Favites*, *Galaxea*, *Montipora*, and *Platygyra*, suggesting that macroalgae may hinder the growth of corals, corresponding to the results that the algal cover has increased. In contrast, coral cover has declined from 2020 to 2022. It is reported that the competition between corals and macroalgae for limited resources (space and light) may lead to reductions in coral growth and survival, which have a serious impact on the structure and function of coral reef ecosystems (Clements et al. 2018; Tanner 1995). Studies have shown that turf and macroalgae often tend to compete better with corals than with crustose coralline algae. However, a phenomenon existed that the coral cover increased in 2023 compared to 2022 on the premise that macroalgal abundance continued to rise, thus elucidating that an increase in macroalgal abundance may not necessarily lead to an increase in coral–algal interactions, which was contrary to most existing studies (Hughes 1994; Hughes 1989).

Several studies have determined that the outcome of coral‐algae interactions depends on a series of factors, including the types of corals and algae involved, the size and growth form of coral communities, and the proportion of macroalgae in contact with the coral (Barott et al. 2012; Bender et al. 2012; Brown et al. 2017; Swierts and Vermeij 2016). Furthermore, research has found that encrusting corals interact most frequently with turf algae, and also compete most successfully with turf algae. While the situation of branched corals is just the opposite and they rarely interact with turf algae and seldom win the competition (Swierts and Vermeij 2016). The species‐specificity can act as the factor influencing the competitive dynamics of coral–algae interactions (Haas et al. 2009). Furthermore, it has been reported that when coral cover is high, interactions between corals themselves are the principal form of competition (Connell et al. 2004). This emphasizes that the frequency of coral–algal interactions is dependent on the abundance of hard coral, not just macroalgal cover, and an increase in coral–algal interactions does not necessarily translate to the degradation of coral reefs. The ecological mechanisms of coral‐algal phase shifts and its environmental driving factors have been extensively studied (McCook et al. 2001; McManus and Polsenberg 2004; Cheal et al. 2010; Christophe 2020), which is the combined effect of multiple factors, including coral mortality, decreased herbivory, and increased nutrients that ultimately lead to the phase shift (Folke et al. 2004; Hughes et al. 2007). A laboratory study conducted in the Great Barrier Reef of Australia examined the competition between a *Lobophora* species and *Porites cylindrica* , and the results showed that an increase in the algal biomass would cause the mortality of coral tissue (Jompa and McCook 2002a, 2002b). And other experimental studies showed that direct algal contact and their lipid‐soluble extracts had bleaching effects on *P. porites* (Rasher and Hay 2010), *Montastraea cavernosa* (Slattery and Lesser 2014), *Acropora muricata* and *Montipora hirsuta* (Vieira et al. 2016). However, more direct experimental evidence is needed to further confirm this conclusion in the future.

## 5 Conclusions

In general, this study revealed that there were 177 species of reef‐building corals belonging to 49 genera and 18 families in the Wenchang Coastal area of Hainan Province through the field investigation of coral reef ecosystems, among which the coral taxa *Favites*, *Porites*, *Galaxea*, and *Montipora* were dominant. The macroalgal species represented by *Lobophora* hinder the growth of corals. The salinity, NO_2_^−^, and NH_4_^+^ were the key environmental drivers that affected the macroalgal abundance. Except for the interactions between corals and algae, the interspecific competition among corals and algae themselves also played an important role in influencing the dynamics of coral–algal interactions. These results would facilitate further understanding of the ecological mechanism of coral–algal phase shifts in Wenchang so as to lay a solid theoretical foundation for relevant departments to scientifically formulate coral reef protection and management.

## Author Contributions

**Yihua Lyu:** conceptualization (equal), investigation (equal). **Yangmei Zhang:** formal analysis (equal), writing – original draft (equal). **Yuqian Li:** formal analysis (equal), writing – original draft (equal). **Youqi Wang:** data curation (supporting), software (supporting). **Zhiqiang Chen:** conceptualization (equal), investigation (equal). **Yingyi Huang:** data curation (equal), software (equal). **Tuanjie Li:** conceptualization (equal), investigation (equal).

## Funding

This work was supported by the Open Fund of Nansha Islands Coral Reef Ecosystem National Observation and Research Station (NSICR23104), the National Key Research and Development Project (2022YFC3106303), and the Provincial and Ministerial‐level research Project: Comparative Study on Natural Restoration and Artificial Restoration of Coral Reefs (YD2203).

## Conflicts of Interest

The authors declare no conflicts of interest.

## Supporting information


**Data S1:** ece372746‐sup‐0001‐DataS1.xlsx.

**Data S2:** ece372746‐sup‐0002‐DataS2.xlsx.

## Data Availability

The raw data used for the analysis of this work are available in Appendix Tables 1–6 in Supporting Information and Tables S1–S11 or by contacting the authors.
